# Baicalin from *Scutellaria baicalensis* blocks respiratory syncytial virus (RSV) infection and reduces inflammatory cell infiltration and lung injury in mice

**DOI:** 10.1038/srep35851

**Published:** 2016-10-21

**Authors:** Hengfei Shi, Ke Ren, Baojie Lv, Wei Zhang, Ying Zhao, Ren Xiang Tan, Erguang Li

**Affiliations:** 1 Medical School and State Key Laboratory of Pharmaceutical Biotechnology, Nanjing University, Nanjing, China; 2 Jiangsu Laboratory of Molecular Medicine, Medical School, Nanjing University, Nanjing, China; 3 College of Life Sciences, Nanjing University, Nanjing, China; 4 Nanjing University of Chinese Medicine, Nanjing, China

## Abstract

The roots of *Scutellaria baicalensis* has been used as a remedy for inflammatory and infective diseases for thousands of years. We evaluated the antiviral activity against respiratory syncytial virus (RSV) infection, the leading cause of childhood infection and hospitalization. By fractionation and chromatographic analysis, we determined that baicalin was responsible for the antiviral activity of *S. baicalensis* against RSV infection. The concentration for 50% inhibition (IC_50_) of RSV infection was determined at 19.9 ± 1.8 μM, while the 50% cytotoxic concentration (CC_50_) was measured at 370 ± 10 μM. We then used a mouse model of RSV infection to further demonstrate baicalin antiviral effect. RSV infection caused significant lung injury and proinflammatory response, including CD4 and CD8 T lymphocyte infiltration. Baicalin treatment resulted in reduction of T lymphocyte infiltration and gene expression of proinflammatory factors, while the treatment moderately reduced RSV titers recovered from the lung tissues. T lymphocyte infiltration and cytotoxic T lymphocyte modulated tissue damage has been identified critical factors of RSV disease. The study therefore demonstrates that baicalin subjugates RSV disease through antiviral and anti-inflammatory effect.

The human respiratory syncytial virus (RSV) infection is a leading cause of acute respiratory tract infections in early childhood^1^. The infection causes flu-like symptoms and is frequently associated with bronchiolitis and pneumonia. It was estimated by the World Health Organization that RSV is responsible for over 33 million new episodes of acute lower respiratory infection in children younger than 5 years^2^. RSV infection is the leading cause of hospitalization of young children with respiratory infections^3^,^4^,^5^. It has also become a significant burden in the elderly among the industrialized countries^3^,^4^,^6^.

RSV is a negative sense, single stranded RNA virus of the *Paramyxoviridae* family that also includes measles, mumps and an assortment of pathogens that cause respiratory tract infections. The transmission of RSV is difficult to prevent since it is easily transmitted by close contact and by unprotected coughing and sneezing. Although virtually all children will have had an infection by the age of two^7^, recurrent infection is very common since the virus has developed an arsenal of strategies to skew host immune response and antiviral immunity^8^,^9^. The virus has the ability to modulate cytokine and chemokine signaling networks, interfere with immune cell function, and antibody response, which possesses an insurmountable challenge to vaccine development. The treatment for RSV infection is limited to treatment of its symptoms since routine use of bronchodilators or antiviral ribavirin has proven to be of no significant benefit, while specific inhibitors are yet to be available^10^,^11^,^12^.

Baicalin is a major component isolated from the radices of *Scutellaria baicalensis* Georgi, an herbal remedy that has been used for over two thousand years in traditional Chinese Medicine for conditions such as viral infection and inflammation. Recent studies demonstrated baicalin with *in vitro* and *in vivo* antiviral activity against influenza viruses^13^,^14^, dengue virus^15^,^16^, enterovirus-71^17^, and Japanese encephalitis virus^18^. The compound targets cell attachment and intracellular replication of H1N1 and H3N2^13^,^14^,^19^. In a preliminary screening study of herbs, Ma and colleagues reported *S. baicalensis* extract has anti-RSV activity^20^. In a patent filing, Zhong *et al*. claimed that ethanol extracts obtained from the genus of Scutellaria had anti-RSV activity^21^. Since no further studies have been reported on the antiviral activity, we decided to test the antiviral activity since RSV is a common pathogen of seasonal diseases. We found that baicalin blocks virus attachment and inhibits virus replication. In a murine RSV infection model, baicalin treatment significantly reduced macrophage and T lymphocyte infiltration to the lung. We also determined the relative content of flavonoids in an extract from *S. baicalensis* and found that baicalin represents the antiviral effect of the radices. Here we report the antiviral effect of baicalin against RSV infection.

## Results

### Cell culture evaluation of *Scutellaria baicalensis* activity against RSV infection

**Figure 1:**
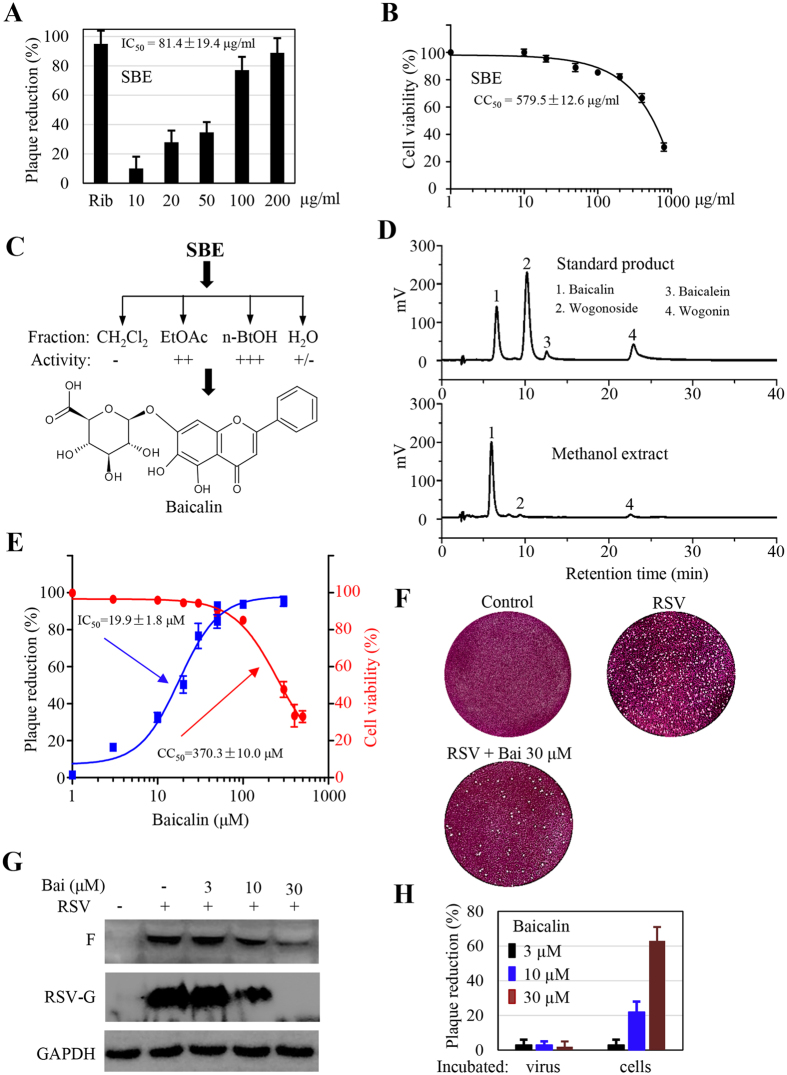
Identification of *Scutellaria baicalensis* antiviral activity in cell cultures. (**A**) Inhibition of plaque formation by an aqueous extract of *S. baicalensis* (SBE). Monolayers of HEp-2 cells in 12-well plates were pretreated in triplicate with SBE at indicated concentrations for 30 min. The cells were then infected with RSV. The effect was determined by counting plaques. The IC_50_ was extrapolated after GraphPad plotting. (**B**) Determination of cytotoxic effect. The cytotoxic effect of SBE was determined by measuring cell viability using MTT method. HEp-2 cells in 96-well plates (3 × 10^3^/well) were treated in triplicate with SBE at indicated concentrations for 3 days. Cell viability was measured colorimetrically. OD_570_ values relative to that of solvent-treated control were plotted for estimation of CC_50_ value. (**C**) Activity-directed fractionation. SBE resuspended in water was extracted with dichloromethane (CH_2_Cl_2_), followed by acetyl acetate, and n-butanol sequentially. After evaporation of solvents, the residues were resuspended and tested at 100 and 500 μg/ml against RSV infection. −: no activity, ++: with activity at 500 μg/ml, +++: with activity at 100 and 500 μg/ml, and +/−: marginal. (**D**) HPLC profiling of flavonoids in ethanol extract from radices of *S. baicalensis*. Upper panel shows the retention time of commercial standards. (**E**) IC_50_ and CC_50_ determination of baicalin, a major component from SBE. (**F**) Typical plates from plaque forming assay. (**G**) Baicalin treatment suppresses RSV F and G protein expression. HEp-2 cells were infected with RSV at 1 MOI in the presence or absence or baicalin at indicated concentrations for 36 hr. RSV-G and F protein expression was detected with specific antibodies. GAPDH was used as a loading control. (**H**) Baicalin does not possess virucidal activity. HEp-2 cells or RSV (in 50 μl medium) was incubated with baicalin at indicated concentrations at 37 °C for 60 min. Baicalin-treated RSV was then diluted by 20-fold and used for infection assays. Data are average +/− SEM of triplicated samples.

To determine the antiviral effect, we performed a quick assay against RSV infection in cell cultures using an aqueous extract from the roots of *S. baicalensis* (SBE). The extract displayed distinct activity against RSV infection under the maximum non-cytotoxic concentrations since there were significantly less plaques in SBE-treated samples (Fig. 1A,B). The 50% inhibitory concentration (IC_50_) was at approximately 81.4 μg/ml in a plaque formation assay.

We next performed a bioassay-directed fractionation tracing the active components. An aqueous extract was fractionated into dichloromethane, acetyl acetate, n-butanol, and water fraction. After evaporation of the solvents, the extracts were reconstituted into DMSO at 1 mg/ml and reevaluated for their antiviral activity. We found the activity was retained and enriched in the n-butanol and, to a less degree, the acetyl acetate fractions (Fig. 1C). The two fractions were pooled and were subjected to chromatographic isolation and purification. Baicalin which was isolated as a major component and 3 other compounds were identified by spectroscopic methods and by comparison with literature reports. The minor components were identified as baicalein, wogonin, and wogonoside, which was consistent with literature report^22^,^23^. Their presence in the roots was further validated by HPLC analysis. As shown in Fig. 1D, baicalin was detected as the predominant flavonoid in the root. Baicalein, wogonoside, and wogonin were detected at significantly less abundance (Fig. 1D).

Since baicalin exhibited the most potent activity against RSV infection in initial screening and represented the most abundant component, its antiviral activity was therefore more thoroughly examined. We first determined its cytotoxic effect by obtaining a half maximal cytotoxic concentration (CC_50_), which was at approximately 370 μM. To obtain an IC_50_ value, monolayers of HEp-2 cells in 6-well plates were treated with baicalin at varying concentrations at 2 hr prior to the infection. During plaque forming assay, baicalin at the corresponding concentrations was supplemented in the overlay medium. We found baicalin treatment significantly reduced the number of plaques (Fig. 1E,F). The IC_50_ was determined at approximately 19.9 μM, a concentration of about 17-fold lower than that of the CC_50_ value. Concomitantly, baicalin treatment resulted in significant reduction in viral protein expression. As shown in Fig. 1G, both RSV-G and F protein expression was substantially reduced in samples treated with 1αnd 30 μM baicalin.

We found the antiviral activity of baicalin was not due to direct virucidal effect since co-incubation of the virus showed no effect on plaque formation (Fig. 1H).

### Baicalin blocks RSV attachment and suppresses RSV replication

**Figure 2:**
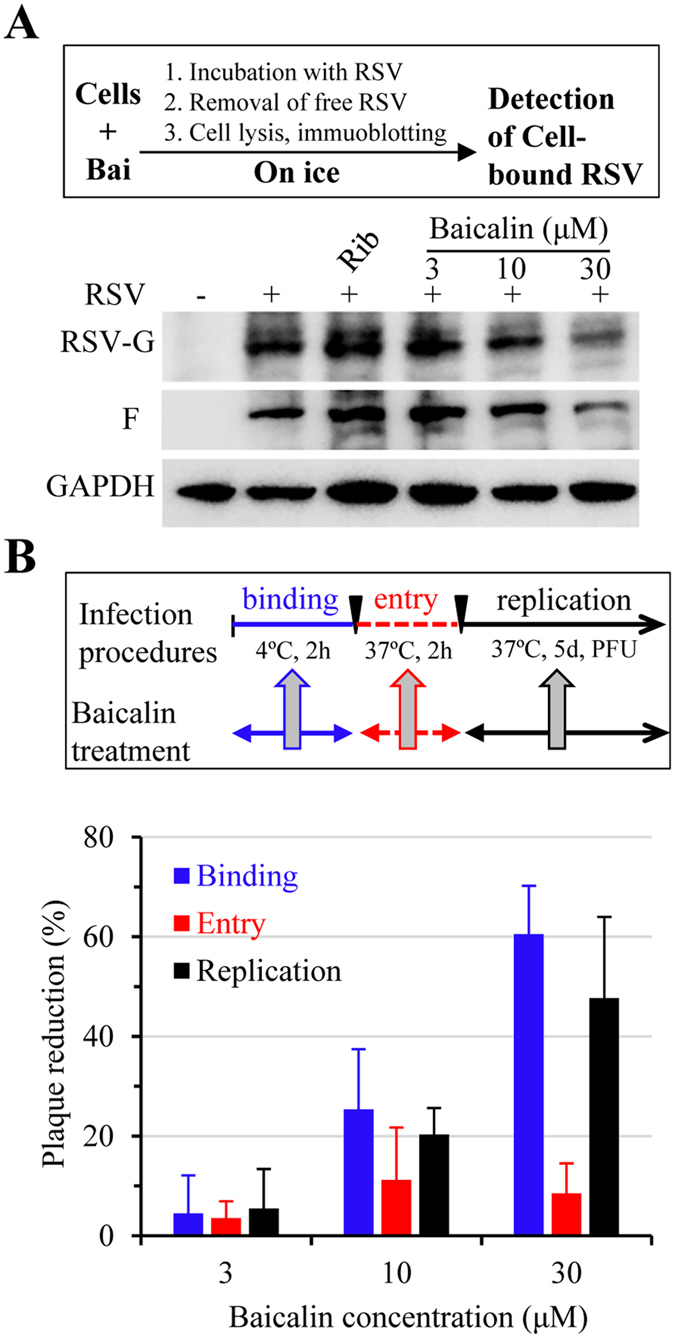
Baicalin targets multi-stages of RSV infection. (**A**) Baicalin blocks RSV binding to HEp-2 cells. Detached HEp-2 cells were un-treated or pretreated with baicalin followed by RSV. After incubation on ice for 90 min, non-bound RSV was removed by centrifugation. Cell-bound virus was detected by immunoblotting analysis for RSV-F and -G proteins. The experiment was performed twice independently. (**B**) Baicalin inhibits RSV infection by blocking virus binding and replication. HEp-2 cells were treated as indicated to test whether baicalin inhibited RSV binding (blue bars), cell entry (red bars), or replication (black bars). The effect was determined by measuring reduction on plaque formations. Data are average +/− SEM of duplicated samples.

Virus infection involves cell attachment, followed by cell entry and subsequent replication. To address whether baicalin affected virus cell attachment, detached HEp-2 cells were incubated on ice with RSV in the presence or absence of baicalin at varying concentrations. The whole process was kept on ice to prevent virus from entering the cells. After 90 min incubation with occasional mixing, the cells were then washed with ice cold medium to remove non-binding virus. The cells were then lysed and used for detection of cell-attached virus by immunoblotting analysis for RSV G and F proteins. We found that cell associated RSV was reduced gradually with the increase of baicalin concentrations, indicating that baicalin had the ability to disrupt RSV attachment to the host cells (Fig. 2A).

Next, we performed an infection assay to substantiate the observation from RSV binding assay. Monolayers of HEp-2 cells in 6-well plates were incubated on ice for 2 hr with RSV (approximately 500–1000 PFU/well) to allow virus attachment in the absence or presence of baicalin at indicated concentrations. Non-attached virus was removed by washing with ice cold medium. Cell-attached RSV was allowed to enter host cells by incubating at 37 °C for 2 hr without baicalin added. At the end of the experiment, the culture medium was removed and was replaced with a culture medium containing 1.5% CMC-Na to allow viral replication and plaque formation without baicalin. As shown in Fig. 2B (blue bars), baicalin treatment during virus binding stage resulted in significant reduction of viral plaque formation, confirming the result from virus binding studies.

We also tested whether baicalin possessed activity against virus cell entry process. In this regard, we first allowed RSV to attach to HEp-2 cells in the absence of baicalin. At the end of the incubation, the medium was removed and was replaced with a culture medium containing varying amount of baicalin. The samples were then moved to 37 °C and were incubated for 2 hr to allow RSV entering host cells in the presence of baicalin (Fig. 2B, red bars). After rinsing off baicalin, the cells were covered with a medium for plaque formation without added baicalin. As shown in Fig. 2B, addition of baicalin during virus cell entry stage only marginally reduced the number of plaques, indicating the compound did not target RSV cell entry for its antiviral effect.

Similarly, we determined whether baicalin suppressed RSV replication. Baicalin was added to the overlay medium after virus binding and cell entry stage. As shown in Fig. 2B (black bars), addition of 10 and 30 μM baicalin during virus replication stage resulted in significant reduction in plaques, indicating the compound also suppressed RSV replication.

### *In vivo* evaluation of baicalin antiviral effect

**Figure 3:**
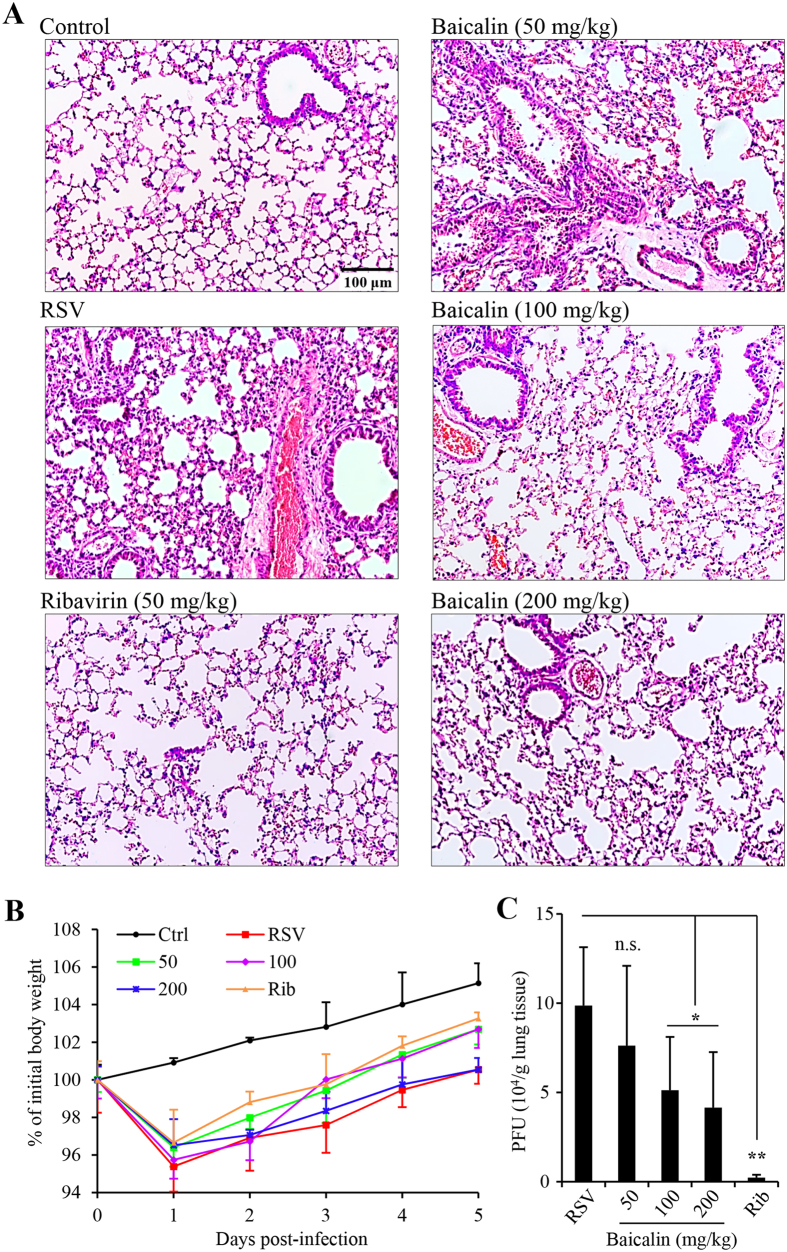
Baicalin treatment ameliorates lung damage and reduces viral load in mice. Female Balb/C mice was infected with RSV (5 × 10^6 ^PFU in 100 μl) by nasal instillation. The animals were randomly grouped (n = 5) and treated started on the next day. Baicalin was delivered by gavage, while ribavirin at 50 mg/kg by intraperitoneal injection daily. The animals were killed on day 5. The right lungs were used for titration of infectious virus and for gene expression studies. The left lung was fixed in 10% formalin and used for histological sectioning. (**A**) H&E stained lung sections. The scale bar represents 100 μm. (**B**) Daily body weight changes as a percentage of weight prior to inoculation. Day 0 refers to time right before inoculation. Data are mean +/− SD (n = 5). (**C**) Viral titers from lung homogenates (n = 3). n.s. represents statistically no significance, *denotes p < 0.05, and **denotes p < 0.01.

We examined the effect of baicalin on RSV infection in a mouse model. Twenty fours after infection by nasal instillation, mice were treated by daily oral administration with baicalin or with antiviral drug ribavirin at 50 mg/kg by intraperitoneal injection (i.p.). The animals were monitored for body weight changes daily. On day 5 following the challenge, the animals were killed and the lungs were harvested for analysis of inflammation and for titration of viral load. As shown in Fig. 3, RSV infection recapitulated previously reported abnormal histology of lung sections. H&E staining showed alveolar walls, and alveolar spaces filled with moderate to severe inflammatory infiltrates of polymorphic mononuclear cells in infected and mock-treated group (Fig. 3A). The lung sections from ribavirin treated group were more closely resembled to that of the uninfected controls except that there were more polymorphonuclear cells (PMNs). Baicalin treatment dose-dependently reversed lung inflammation and inflammatory cell infiltration. The effect of baicalin on mouse body weight changes was less significant (Fig. 3B), while baicalin was able to moderately reduce viral load (Fig. 3C).

### Baicalin reduces lymphocyte infiltration and suppresses proinflammatory gene induction

**Figure 4:**
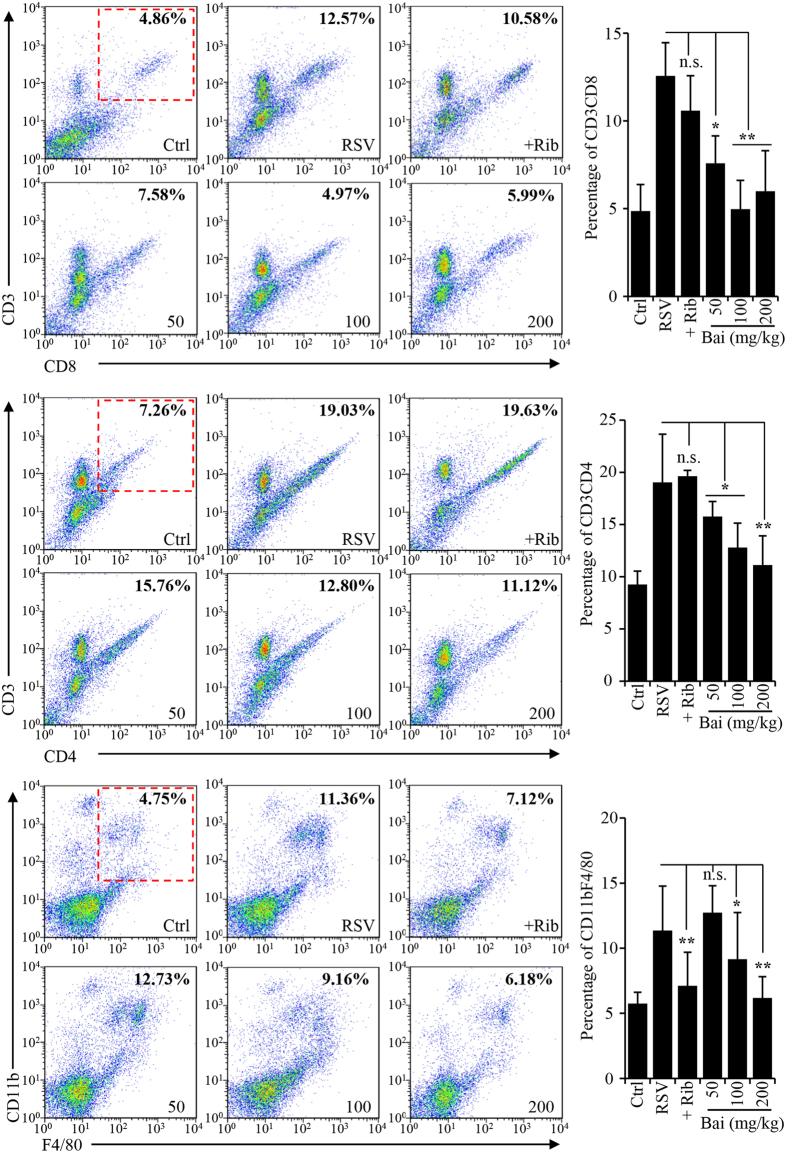
Flow cytometry analysis of inflammatory cells in the lung tissues. Single-cell suspensions were prepared from lung tissues as described in materials and methods. The cells were stained with APC-labeled anti-F4/80 and FITC-labeled anti-CD11b, APC-labeled anti-CD3 and PE-labeled anti-CD8, or with APC-labeled anti-CD3 and FITC-labeled anti-CD4. FlowJo7.6.1 software (TreeStar, Ashland, OR) was used for data analysis. The bar graphs on the right represent quantitative measure of positively stained cells. Data are average +/− SEM (n = 3). n.s. represents statistically no significance, *denotes p < 0.05, and **denotes p < 0.01.

Lung inflammation was also investigated by quantitatively determining inflammatory cell infiltration using FACS. As shown in Fig. 4, RSV infection promoted T lymphocyte and macrophage infiltration. Baicalin treatment resulted in marked reduction of CD4 and CD8 T lymphocytes and macrophages in the lung tissues. Although ribavirin treatment resulted in more profound reduction in viral load compared to that in baicalin treated groups, interestingly, both CD4^+^ and CD8^+^ cells remained at relatively high levels in the ribavirin-treated animals.

**Figure 5:**
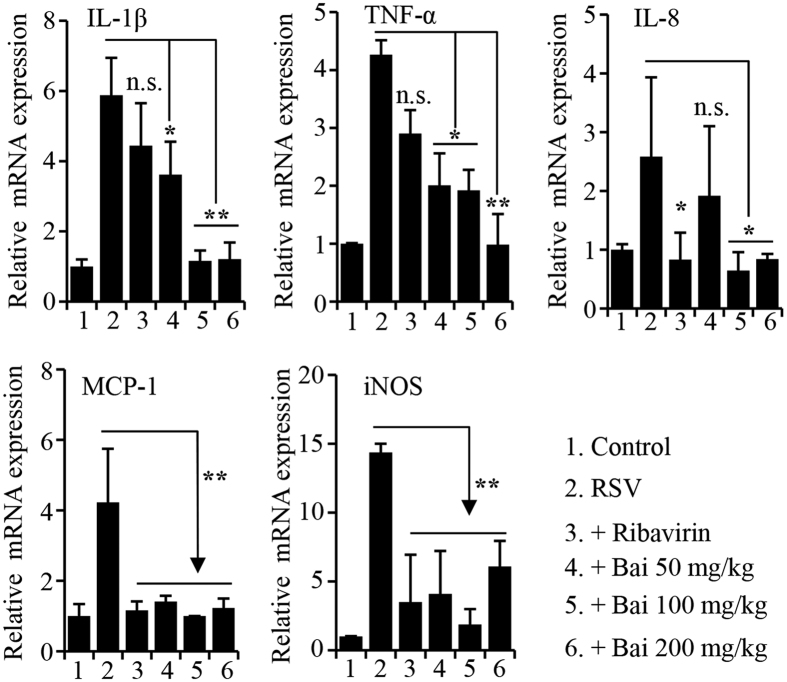
Proinflammatory gene expression determined by real-time PCR. RNA from mouse lung tissues was isolated. Quantitative PCR was performed on the Bio-Rad C1000 real-time PCR system using SYBR green Master Mix reagent (Bio-Rad). The data were analyzed using the 2^−ΔΔCt^ formula. GAPDH was used as an internal for determination of gene expression. Three animals were used from each group (n = 3). n.s. represents statistically no significance, *denotes p < 0.05, and **denotes p < 0.01.

We also performed qPCR studies to determine proinflammatory gene expression. Consistent with results from histological studies, there was significant reduction in proinflammatory gene expression (Fig. 5). Those results, together, demonstrated baicalin, a major component of *S. baicalensis*, suppresses RSV infection by targeting virus attachment, intracellular replication, as well as by ameliorating proinflammatory response.

### Baicalin represents *Scutellaria baicalensis* antiviral activity

**Figure 6:**
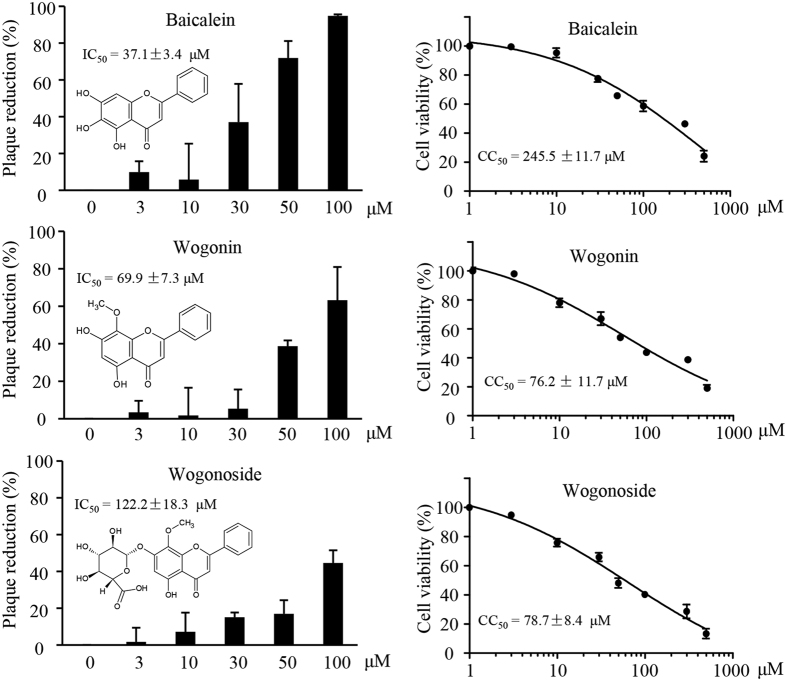
Evaluation of antiviral activity and cytotoxic effect of baicalein, wogonin, and wogonoside. HEp-2 cells were used for antiviral and for cytotoxicity assays. IC_50_ and CC_50_ data were extrapolated from GraphPad plotting. Data are average +/− SEM of triplicated samples. The experiments were performed twice independently.

The radices of *S. baicalensis* contain liposaccharides, terpenoids, and flavonoids. In addition to baicalin as the major component, we also identified baicalein, wogonin, and wogonoside as minor components. To determine whether baicalin was responsible for the antiviral activity of *S. baicalensis*, we therefore compared the antiviral activity of baicalin to those minor components using commercially purchased compounds. Compared to that of baicalin, those compounds showed relatively weak to moderate antiviral activity. The IC_50_ values for baicalein, wogonin and wogonoside were measured at 37, 122, and 69 μM, respectively, very close to the CC_50_ values in the cases of wogonin and wogonoside (Fig. 6). In view of those results and the relative abundance of the compounds, we concluded that baicalin represented *S. baicalensis* anti-RSV activity.

Together, the results demonstrated that baicalin, a major active component of *S. baicalensis*, inhibits RSV infection by blocking RSV cell attachment and viral replication. The compound improves disease condition by suppression of lymphocyte infiltration and proinflammatory cytokine expression.

## Discussion

RSV is a leading cause of lower respiratory tract disease and bronchiolitis in children worldwide. Despite of decades of effort, there is no safe and efficacious RSV vaccines. The selection for treatment commonly includes symptomatic supportive like bronchial dilation agents, while the selection of antiviral therapy is limited to ribavirin as supplemental. Herbal medicines have demonstrated therapeutic efficacy for symptoms of viral infection and inflammation although the underlining mechanisms are not clear. In this study, we identified baicalin as an active component of *S. baicalensis* antiviral activity. Baicalin blocks RSV infection in several aspects, including blocking cell attachment and intracellular replication. Baicalin treatment suppresses inflammatory cell infiltration, alleviates lung injury.

T cell response has a critical role in antiviral immunity and the resolution of an infection. The host senses RSV invasion with pattern recognition receptors, leading to increased cytokine production, inflammatory cell influx, and subsequently mucus production and reduced lung function^8^. Both CD4^+^ and CD8^+^ lymphocyte subsets were involved in terminating RSV replication after primary infection^24^. CD8^+^ T cells have been shown to be detrimental in RSV-infected infants. In fact, they have been found to coincide with convalescence in infants with severe disease^25^. Inhibition of CD8^+^ T lymphocyte function attenuates RSV-enhanced allergic inflammation^26^. There is a Th1 to Th2 imbalance of CD4^+^ T cell responses in young infants undergoing the disease, which may be a contributing factor in lingering coughing and wheezing common in infants with severe RSV disease^27^,^28^,^29^. In addition to reduction of lung injury and a moderate reduction of viral titers, we observed a decrease in both CD4 and CD8 cells in baicalin treated groups. Although viral loads in ribavirin-treated group were close to non-detectable levels, both CD4 and CD8 cells remained at relatively high levels in this group. Different sets of immune cells persist in the lungs even after viral clearance^30^,^31^. Both clinical and animal studies consistently shown that both CD4^+^ and CD8^+^ cells are immunopathogenic to the host and may have contributed to the augmented disease seen in patients^32^, indicating that the host has difficulty in resolving immune cell response to RSV infection. Baicalin is known to have immunomodulatory activity against autoimmune diseases^33^,^34^,^35^. It is likely that baicalin exhibits antiviral effect against RSV infection not only by blocking viral infection processes but also by restoring a skewed immune system.

*Scutellaria baicalensis* Georgi is one of the most widely used medicinal plants for the implications of inflammation, tumor, viral and bacterial infections according to Chinese Pharmacopoeia. Baicalein, wogonin, and baicalin are among the main compounds with biological activities^20^,^23^. Although baicalin, wogonin and oroxylin A were also reported with anti-RSV activity^20^, wogonin showed weak activity compared to that of baicalin. We performed HPLC profiling and fractionation studies and identified baicalin as a component responsible for the anti-RSV effect.

RSV infection promotes rapid ROS production that in turn induces cytokine and chemokine production^36^,^37^,^38^,^39^,^40^. Uncontrolled production of ROS exacerbates lung inflammation and tissue damage^41^,^42^. Accordingly, antioxidants treatment ameliorates RSV-induced pulmonary inflammation and disease^39^,^43^. Baicalin is a bioflavonoid that is known to have antioxidant activity. In this study, we found that the IC_50_ values of compounds with similar structure features varied significantly, suggesting that its antiviral activity is not likely linked to the antioxidant property.

Baicalin possesses antiviral activities against HIV, influenza, and Dengue viruses with an assortment of mechanisms of action. We found that baicalin blocked RSV infection through inhibition of cell attachment and intracellular replication, a mechanism that is also shared against influenza and dengue infection^13^,^15^. Other mechanisms include baicalin inhibition of autophagy activity and innate immune responses^19^,^44^. Baicalin is a potent immune modulator in other disease models^35^. We found mice treated with baicalin had more speedy resolution of T lymphocyte infiltration. It is interesting to determine whether cellular factors are involved in the *in vivo* anti-RSV effect of baicalin.

## Materials and Methods

### Cells and viruses

Human epidermoid cancer cell line (HEp-2, CCL-23) and African green monkey kidney epithelial cell line (Vero, CCL-81), originally obtained from ATCC (Manassas, VA, USA), were purchased from Cell Bank of Chinese Academy Sciences (Shanghai, China). Human lung adenocarcinoma epithelial cell line (A549, CCL-185) was purchased from the ATCC. The cells were cultured in DMEM (Dulbecco’s modified Eagle’s medium, high glucose, Life Technologies, Carlsbad, CA, USA), supplemented with 10% heat inactivated fetal bovine serum (FBS), *L*-glutamine, nonessential amino acids, and sodium pyruvate at 37 °C in a humidified atmosphere with 5% CO_2_. Human RSV strain A2 was propagated in A549 cells as previously described^45^. Because RSV retains a close association with the host cell membrane and has a tendency to aggregate during centrifugation procedures, the stock of crude supernatant was used after a titer was determined.

### Reagents and antibodies

Baicalin (STA-18606044), baicalein (STA-18706043), wogonin (STA-17106063) were purchased from Nature Standard (Shanghai, China). Wogonoside was purchased from DASFBio (Nanjing, China). Antibody to RSV F protein (sc-101362) was obtained from Santa Cruz Biotechnology (Dallas, TX), G protein (ab94966) was obtained from Abcam. GAPDH antibody (MB001) was obtained from Bioworld Technology (Minneapolis, MN). HRP-conjugated secondary antibodies were purchased from Bio-Rad, and Alexa Fluor-conjugated antibodies were from BD.

### *In vitro* cytotoxicity assay

We first determined the cytotoxic effect of baicalin, baicalein, wogonin and wogonoside by measuring cell viability on HEp-2 cells. Briefly, HEp-2 cells (3 × 10^3^ were seeded into 96-well plate. A test compound dissolved in DMSO was diluted with fresh medium and tested in triplicates on HEp-2 cells. DMSO at a final concentration of 0.1% or less was included as mock-treated control. The cells incubated for 72 hr, at which time 20 μl MTT at 0.5 mg/ml was added and incubated for another 4 hr. Formazan was dissolved in 100 μl DMSO and measured at 570 nm on a microtiter plater reader. The OD readings relative to mock treated samples were plotted and CC_50_ was extrapolated using GraphPad Prism (San Diego, CA, USA).

### Plaque assay

HEp-2 cells in 12 or 6-well plates were incubated with 300–500 μl medium containing RSV (approximately 200–500 PFU) for 60 min or as stated with occasional shaking. The inoculum was removed and 2–3 ml culture medium containing 1.5% sodium carboxyl methyl cellulose (CMC-Na, medium viscosity, C4888, Sigma) was added. The overlay contains DMEM with 5% FBS, and a test compound at indicated concentrations. DMSO at 0.1% or less was used as solvent control. The plates were incubated at 37 °C for 5–7 days. At which time, the cells were fixed with 3% paraformaldehyde in PBS, then stained with 0.1% crystal violet solution for plaque enumeration. A concentration that reduces plaque numbers by 50% was defined as IC_50_. The experiment was performed three times independently.

### Virus binding assay

To determine RSV binding, HEp-2 cells were detached by treatment with 2 mM EDTA (pH 7.6). The cells were washed with ice cold DMEM three times and then were resuspended in DMEM supplemented with 0.5% bovine serum albumin (1 × 10^7 ^cells/ml). The cells were aliquoted (100 μl/sample) and were untreated or incubated with baicalin at 3, 10 and 30 μM concentrations. As a control, we used ribavirin at 50 μg/ml since the compound is a known antiviral agent that blocks nucleoside synthesis. After pre-incubation with baicalin for an hour, RSV stock (about 30 PFU/cell) was added to the samples. The cells kept on ice for 90 min with occasional tapping. At the end of the incubation, the cells were washed with ice cold DMEM (at 1000 × g for 5 min) to remove non-bound virus. The cell pellets were lysed with 1x Laemmli sampling buffer and used for the detection of cell-bound RSV by immunoblotting for the RSV-G and F proteins.

### Western blotting assay

Cells were harvested in a lysis buffer containing 1% NP-40, 150 mM NaCl, 50 mM Tris-HCl (pH 7.4), and a cocktail of protease inhibitors (Roche). After removal of cellular debris, soluble proteins were separated by SDS-PAGE and transferred to a polyvinylidene difluoride (PVDF) membrane (Millipore). The proteins were detected by incubation with a primary antibody, followed by horseradish peroxidase-conjugated secondary antibody, and the ECL reagent kit (Pierce). The images were captured using the Clinx ChemiScope imaging system (Shanghai, China).

### Pulmonary infection in mouse model

Animal studies were approved by Medical School for Animal Use and Care Committee of Nanjing University in accordance with the guideline of the US NIH. Female Balb/C mice, 4–6 weeks old, were purchased from Model Animal Research Center of Nanjing University. The animals were kept in a specific pathogen-free environment and fed with food and water *ad libitum*.

Mouse model of RSV pulmonary infection was established by nasal instillation of RSV (5 × 10^6 ^PFU in 100 μl) to lightly sedated mice (5 per group)^46^. On the next day, the animals were treated daily with a vehicle (control group), baicalin at 50, 100 or 200 mg/kg, or ribavirin at 50 mg/kg by intraperitoneal injection. The animals were monitored daily and were killed on day 5. The lungs were removed, weighed, and the right lung was used for titration of infectious virus and for gene expression studies. The left lung was used for flow cytometry analysis of cell infiltration. In parallel, the left lung was submerged in 10% formalin for fixation for 24 hr and subsequent histological sectioning.

### Flow cytometry analysis for macrophages and T lymphocyte infiltration

Single-cell suspensions were prepared from lungs by cutting into small fragments and digested for 45 min at 37 °C with type I collagenase (3 mg/ml) and DNase I (30 μg/ml) in RPMI 1640 medium. Digested lungs were mechanically disrupted by passage through a sterile strainer (100 μm, Falcon, BD Biosciences) using the flat portion of a plunger from a 3-ml syringe followed by an additional 40 μm strainer (Falcon, BD Biosciences). Red blood cells were lysed with 150 mM NH_4_Cl, 10 mM KHCO_3_ and 0.1 mM EDTA. After washing with ice cold PBS, the cells were collected and resuspended in PBS, stained with APC-labeled anti-F4/80 and FITC-labeled anti-CD11b, APC-labeled anti-CD3 and PE-labeled anti-CD8, or APC-labeled anti-CD3 and FITC-labeled anti-CD4. The samples were analyzed on FACS Calibur (BD Biosciences). FlowJo7.6.1 software (TreeStar, Ashland, OR) was used for data analysis.

### Real-time PCR

RNA from mouse lung tissue was isolated using TRIzol reagent (Life Technologies) and reverse transcribed. Quantitative PCR was performed on the Bio-Rad C1000 real-time PCR system using SYBR green Master Mix reagent (Bio-Rad). GAPDH was used as an internal control for normalization. The data were analyzed using the 2^−ΔΔCt^ formula. The sequences of the primers for mouse gene expression are listed (forward and reverse):

IL-1β: 5′-CTCGTGCTGTCGGACCCAT and 5′-CAGGCTTGTGCTCTGCTTGTGA;

TNF-α: 5′-ATCCGCGACGTGGAACTGGC and 5′-CCATGCCGTTGGCCAGGAGG;

IL-8: 5′-CGGCAATGAAGCTTCTGTAT and 5′-CCTTGAAACTCTTTGCCTCA;

MCP-1: 5′-AGTTAACGCCCCACTCACCT and 5′-TCCTTCTTGGGGTCAGCACA;

iNOS: 5′-AAGTCCAGCCGCACCACCCT and 5′-GTCCGTGGCAAAGCGAGCCA;

GAPDH: 5′-ATCTCCGCCCCTTCTGCCGA and 5′-CCACAGCCTTGGCAGCACCA.

### Plant samples and HPLC determination of flavonoid contents in the radix of *Scutellaria baicalensis*

Dried roots of *S. baicalensis* Georgi was purchased from a drug store in Nanjing. The plant material was authenticated by in-store pharmacist and confirmed by HPLC method following the protocol given in Chinese Pharmacopeia. An extract from the radixes of *S. baicalensis* was prepared and chemical contents were determined following a reported procedure^22^ on a Survit STI-501Plus HPLC system (Hangzhou, China) using a Hypersil Gold C8 column (150 × 4.6 mm, particle diameter of 5 μm, Thermo Fisher). The mobile phase was 18% methanol, 25% acetonitrile, and ultra-pure water with 0.2% acetic acid. The flow rate was set at 0.8 ml per min and the UV detector was set at 275 nm.

### Statistical analysis

Data were analyzed with GraphPad Software (San Diego, CA). Statistical significance of differences was assessed with Student’s t test for two groups or one-way ANOVA for multiple groups. *P* < 0.05 was considered significant. All independent parameters are mean ± standard error of mean (SEM) of results in at least 3 independent experiments unless otherwise stated.

## Additional Information

**How to cite this article**: Shi, H. *et al*. Baicalin from *Scutellaria baicalensis* blocks respiratory syncytial virus (RSV) infection and reduces inflammatory cell infiltration and lung injury in mice. *Sci. Rep.* **6**, 35851; doi: 10.1038/srep35851 (2016).
